# Characteristics of patients who are admitted with or acquire Pressure Ulcers in a District General Hospital; a 3 year retrospective analysis

**DOI:** 10.1002/nop2.50

**Published:** 2016-03-16

**Authors:** Peter R. Worsley, Glenn Smith, Lisette Schoonhoven, Dan L. Bader

**Affiliations:** ^1^ Skin Health and Continence Technology Research GroupClinical Academic FacilityFaculty of Health SciencesUniversity of SouthamptonSouthamptonSO16 6WDUK; ^2^ Nutrition and Tissue Viability Service OfficeTop Floor GMO officesNorth BlockSt Mary's HospitalParkhurst RoadNewportIsle of WightPO30 5TGUK; ^3^ NIHR CLAHRC WessexUniversity of SouthamptonSouthamptonSO17 1BJUK

**Keywords:** Community acquired, hospital acquired, pressure ulcer, prevalence

## Abstract

**Aim:**

The study aimed to characterize demographic and clinical practice factors associated with community (CAPU) and hospital acquired pressure ulcers (HAPU).

**Design:**

A comparative retrospective evaluation of pressure ulcer data, collected from a district general hospital.

**Methods:**

Demographic and pressure ulcer related data were collected from patients at risk of developing a pressure ulcer, collated by a single observer using a standardized tool. Comparisons were made within and between patient groups (no PU, CAPU and HAPU).

**Results:**

CAPU and HAPU patient groups were significantly (*P *<* *0·001) older, had extended lengths of hospital stay and were less likely to be provided quickly with a pressure relieving support surface than those with no PU. HAPU patients had a longer length of stay and a higher proportion of heel PUs compared to CAPU.

## Introduction

There is a growing ageing population living with complex multimorbidities (Smith *et al*. 2012). As a consequence these individuals often have impaired mobility and are supported for prolonged periods in a bed or chair (Brown & Flood 2013). In these positions, they are exposed to loads which can lead to localized compromise of soft tissues, resulting in their breakdown and the development of chronic wounds, typically termed pressure ulcers (PUs) (National Pressure Ulcer Advisory Panel, European Pressure Ulcer Advisory Panel and Pan Pacific Pressure Injury Alliance, 2014). PUs negatively impact on patients' rehabilitation and quality of life (Spilsbury *et al*. 2007). Despite the increased recent attention within the health services, their incidence rate remains unacceptably high (Gallagher *et al*. 2008). Indeed, it is estimated that European healthcare providers each spend between 1‐4% (€1·9‐2·9 billion) of their total budget per year on PU prevention and treatment (Severens *et al*. 2002, Dealey *et al*. 2012). A more recent estimate of the annual costs in the United States is US$ 9·1‐11·6 billion (Agency for Healthcare Research and Quality (AHRQ), 2011), a value that will inevitably increase with an ever ageing population. Patients with reported pressure ulcers in the hospital setting include those who are admitted with a PU acquired in the community (CAPU), and those who acquire a PU during their hospital stay (HAPU) (VanGilder *et al*. 2009). Prevalence rates of PUs among inpatients in hospital settings were estimated at 12·1%, 8·9%, 11% and 10·2% in Belgium, France, Germany and the UK, respectively, of which 40‐59% are HAPUs (Lahmann *et al*. 2006, Barrois *et al*. 2008, Phillips & Buttery 2009, Vanderwee *et al*. 2011). The prevalence rates of CAPUs are particularly high in long‐term care settings such as nursing homes, with prevalence figures ranging from 8·8‐53·2% (Moore & Cowman 2012).

## Background

Although the problem of PUs is widely acknowledged in the healthcare sector it has only recently gained importance in political terms. The political focus is due, in part, to the emerging litigation burden to healthcare providers, which is predicted to increase due to both general societal trends and changes in the law, leading to investigation of severe pressure ulcers by government agencies to detect institutional and professional neglect of vulnerable adults (Department of Health, 2010). This has led to the current interest in determining the onset of the PU (CAPU vs. HAPU) in hospitalized patients. A recent systematic review evaluated the risk factors associated with PU development and found that mobility/activity, perfusion (including diabetes) and skin/pressure ulcer status were the primary predictors (Coleman *et al*. 2013). In addition, several European studies have shownassociations between PU risk and the provision of support surfaces, nutritional status, urinary incontinence, cognitive impairment, low serum albumin length of hospital stay and the frequency/quality of risk assessments (Oot‐Giromini 1993, Keelaghan *et al*. 2008, EPUAP‐NPUAP, 2009, Gunningberg *et al*. 2011, 2013). These factors have been reported to be associated with both CAPU and HAPU. Evidence, however, suggests the impact of HAPU on length of stay is more pronounced compared to CAPU, but this research was limited to patients over the age of 75 years (Theisen *et al*. 2012). Indeed, while the demographic and clinical practice factors may be similar for both groups of patients, the impact of the pressure ulcer on their hospital stay and readmission rates may vary. There is clearly a need to further investigate the differences between CAPU and HAPU patients across a wider hospital population.

This study aims to characterize demographic and clinical practice factors associated with community (CAPU) and hospital acquired pressure ulcers (HAPU). In particular, the study evaluated the patient demographics and key clinical outcomes including the length of hospital stay, readmissions, the provision of pressure redistributing equipment and the monitoring of pressure ulcer risk.

## The study

### Design

Retrospective data were collected in a District General Hospital on an island off the south coast of the UK. It serves a predominantly rural population, a significant proportion (25%) of which are over 65 years of age. The hospital has orthopaedic, surgical and medical specialities and also offers facilities for long term rehabilitation. Patients who require complex surgical medical or orthopaedic support are transferred to nearby specialist centres on the UK mainland.

### Method

All patients admitted to the District General Hospital over 41 months between 2007–2010 were eligible for analysis. Throughout their hospital stay, data were collected by a single observer (GS) using a standardized reporting form to record their risk status and, where present, the location and category of any pressure ulcers. Where patients were readmitted multiple times, the first record of their hospital stay was included for analysis and their subsequent re‐admissions were only documented. Clinical records were collated from all who had a Waterlow Risk assessment score of above 10 at any point during their hospital stay (defined as at risk of a pressure ulcer). Those who did not exceed this risk threshold throughout their hospital stay or did not have a PU present were not included in the analysis. Patients were assessed by a registered nurse within 24 hours of being admitted to hospital, where it was determined that they either presented with a pressure ulcer on admission (CAPU), or had no pressure ulcer present. Patients were excluded if reporting was not conducted by the primary observer (GS), to ensure data consistency. If data were missing, patients were also omitted from the analysis. The tissue viability reporting forms captured information regarding:

- The location from which the patient was admitted
- Date and time of admission
- Specific information regarding;
  1. Date of the Waterlow risk assessment.
  2. Maximum Waterlow score
  3. Site and category of pressure ulcer, where present, using the EPUAP classification system involving categories 1‐4 (EPUAP‐NPUAP 2009)
  4. Time at which a pressure redistributing support surface was obtained
  5. Discharge location or mortality
  6. Readmission rates over the 41 month period

In addition, data from the hospitals central electronic resource, including age, gender and type of admission were used for analysis.

### Analysis

Data from the patient admissions were categorized into three distinct groups, namely;

1. Patients who were at risk (Waterlow >10) during their admission, but did not develop a Pressure Ulcer (NoPU)
2. Patients who had a pressure ulcer on admission, i.e. obtained in the community setting which could include the private home, residential care or nursing home (CAPU)
3. Patients who acquired a pressure ulcer in hospital (HAPU)

Data were collated using a custom software code in Matlab (Mathworks, USA). Key patient demographics and inpatient clinical data were presented using descriptive statistics. To identify trends between the three groups (no PU, CAPU and HAPU) and their respective pressure ulcer severities (categories 1–4), a one way anova test with Tukey *post hoc* analysis was performed for continuous variables, a Mann–Whitney *U*‐test for ordinal scale variables and a Chi‐square test for categorical variables.

### Ethics

Institutional ethics was approved for the study (REC FOHS‐6097), with approval from the Research and Governance Office of the hospital acquired prior to data analysis.

## Results

### Patient demographics

The demographics of the 46,254 patients admitted to the general district hospital reflected the ageing population of the local community, with a mean age of 56·6 years. Of these patients, 6516 (14%) were considered to be at risk of developing a PU presenting with a maximum Waterlow score greater than 10 at some point during their hospital stay. These patients were distributed within the three PU sub‐groups (Table 1), each of which are described separately.

**Table 1:** *Description of patients who were at risk but did not acquire a pressure ulcer (No PU), had a community acquired pressure ulcer (CAPU) and those who developed a pressure ulcer during admission (HAPU)*

No. of Patients		% of PUs	Age (years) mean ± SD	Male (%)	Length of stay (days) median (range)	Peak Waterlow mean ± SD	Mattress within 24hr^a^	Intervals between Risk Assess (days) mean ± SD	Location number (percentage)				
									Sacrum	Heel	Buttock	Elbow	Other
No PU	3851	NA	74 ± 13.0	44	4(0‐229)	15.2±4.2	98%	3.7 ±5.9	NA	NA	NA	NA	NA
Category 1	CAPU (n=916)	70%	79.0 ± 11.5	43	5 (1‐235)	18.2±4.8	89%	3.5 ±4.8	650 (71%)	46 (5%)	211 (23%)	9 (1%)	3 (0%)
	HAPU (n=696)	50%	80.4 ± 11.6	44	10 (1‐186)	20.6±5.3	80%	4.2 ±5.7	362 (52%)	244 (35%)	21 (3%)	42 (6%)	28 (4%)
Category 2	CAPU (n=238)	20%	80.9 ± 12.0	44	7(1‐138)	21.4±5.1	73%	4.3 ±5.5	183 (77%)	19 (8%)	26 (11%)	5 (2%)	6 (3%)
	HAPU (n=510)	36%	80.6 ± 11.1	45	11(1‐169)	20.9±4.4	72%	4.3 ±7.2	332 (65%)	97 (19%)	36 (7%)	15 (3%)	31 (6%)
Category 3	CAPU (n=96)	8%	82.3 ± 12.4	33	9(0‐126)	24.4±4.8	34%	4.6 ±4.4	72 (75%)	4 (4%)	12 (12%)	4 (4%)	5 (6%)
	HAPU (n=160)	12%	82.0 ± 9.6	45	18(0‐205)	23.4±3.5	54%	5.3 ±7.0	107 (67%)	30 (19%)	8 (5%)	3 (2%)	11 (7%)
Category 4	CAPU (n=20)	2%	76.1 ± 12.5	20	7(1‐87)	25.7±3.2	59%	5.0 ±8.9	15 (77%)	4 (19%)	0 (0%)	0 (0%)	1 (3%)
	HAPU (n=32)	2%	73.9 ± 13.2	58	15(1‐193)	26.9±2.9	47%	4.2 ±3.7	12 (37%)	15 (48%)	2 (5%)	2 (5%)	1 (3%)
All Categories	CAPU (n=1267)		79.6 ± 11.7	42	6(1‐235)	18.9 ±5.1	81%	3.7 ±5.0	920 (73%)	63 (6%)	251 (19%)	18 (1%)	15 (1%)
	HAPU (n=1398)		80.5 ± 11.3	45	11(1‐205)	21.0 ±5.0	73%	4.3 ±6.4	813 (58%)	376 (28%)	67 (5%)	62 (4%)	71 (5%)

a Patient given a pressure redistributing mattress within 24 hours of being deemed at risk of a pressure ulcer (Waterlow >10)

#### Patients who were at risk and did not acquire a pressure ulcer

Of the total number of patients, 3851 (8·3%) were at risk but did not acquire a PU at any point during their stay (Table 1). These patients had a mean age of 74 (sd 13·2) years and a median length of hospital stay of 5 days (range 1‐229). This group had an average Waterlow score of 15·2 (sd 4·2) and the majority (n = 3774 or 98%) of these patients received a pressure redistributing mattress within 24 hours of being at risk of developing a pressure ulcer. Of this group of patients, 2231 (58%) were readmitted to the hospital at least two times during the 41 month period. The vast majority (n = 3581 or 93%) were admitted from home, with the remainder being admitted from residential care (n = 193 or 5%) or nursing homes (n = 770 or 2%). Most of these patients attended, the hospital for an emergency admission (n = 2657 or 69%) as opposed to an elective procedure (n = 1194 or 31%).

#### Patients who presented with a Pressure Ulcer on Admission (CAPU)

**Figure 1:**
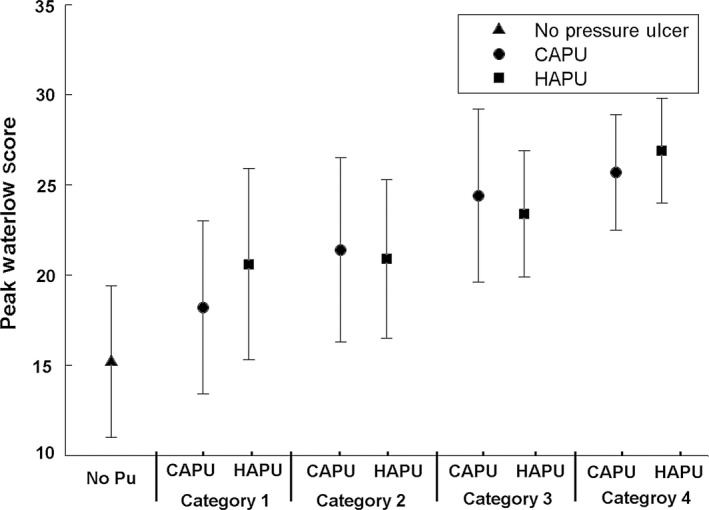
Peak Waterlow scores (mean, standard deviation) from patients with no pressure ulcers, hospital acquired pressure ulcers (HAPU) and community acquired pressure ulcer (CAPU) groups. Results are shown for each category of pressure ulcer (1–4) for the CAPU and HAPU groups.

**Figure 2:**
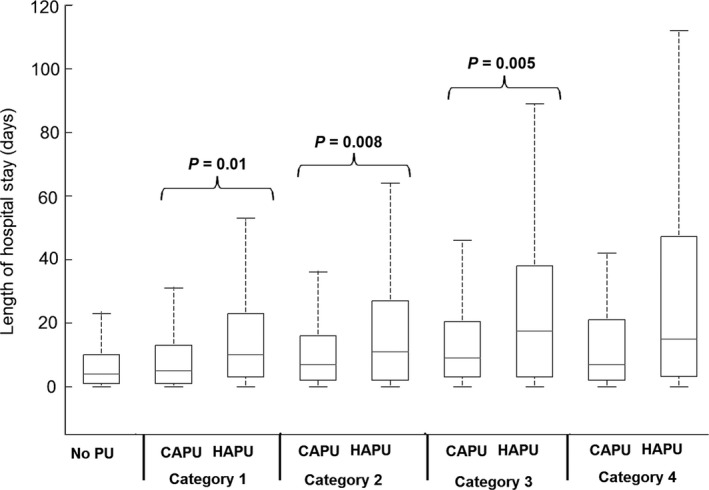
Length of hospital stay (median, inter‐quartile range box and whisper plots) from patients with no pressure ulcers, hospital acquired pressure ulcers (HAPU) and community acquired pressure ulcer (CAPU) groups. Results are shown for each category of pressure ulcer (1–4) for the CAPU and HAPU groups.

There were 1267 patients presenting with one or more PUs on admission (CAPU). Of these patients, 262 had multiple PUs (between 2‐7), which resulted in a total CAPU count of 1473. Patients who were admitted with a CAPU had a mean age of 80 (sd 12) years and a median hospital length of stay of 6 days (range 1‐235). Of the reported CAPUs, the majority were category 1 and 2, representing 70% (n = 916) and 20% (n = 238) of the total respectively (Table 1). Although with increasing severity of CAPU there was an associated increase in the maximum Waterlow scores (Figure 1), there were no corresponding changes in the length of hospital stay (Figure 2). CAPU location did not differ significantly across the categories, with the sacral region demonstrating the highest proportion (71‐77%). The majority (n = 1025 or 81%) of CAPU patients received a pressure redistributing mattress within 24 hours, although this number varied, for example only 35% (34/96) of category 3 CAPU patients received a pressure redistributing device within this time period. This patient group was risk assessed using the Waterlow score at mean intervals of approximately 4 (sd 5) days during their hospital admission. A high proportion (n = 976 or 77%) of the CAPU group was re‐admitted to hospital within the 41 month study period. In addition, the majority of the group were admitted from private homes (n = 1026 or 81%) and were emergency admissions (n = 1038 or 82%).

### Patients with a hospital acquired pressure ulcer (HAPU) `

A total of 1398 patients acquired a pressure ulcer during their hospital stay. Of these patients, 426 (30%) had multiple PUs at different locations on the body (2‐6 different PUs). This resulted in 1848 different pressure ulcers in this sub‐group. These HAPU patients had a mean age of 81 ± 11 years and a median length of hospital stay of 11 days (range 1‐212 days). Of the reported HAPUs, the majority were category 1 and 2 ulcers, representing 50% (n = 696) and 36% (n = 510) of the total number respectively (Table 1). The patients who developed a category 3 or 4 pressure ulcer generally exhibited a longer length of stay and an increased Waterlow score relative to those with less severe PU categories (Figures 1 & 2). The mean time interval between risk assessments was approximately 4 SD 6 days and 73% (n = 1021) of HAPU patients received a pressure redistribution mattress within 24 hours of judged to be at risk of developing a PU (Waterlow score >10). Over all PU grades, 58% (n = 811) were located at the sacrum and 28% (n = 319) at the heels. However, the later site was associated with a higher proportion of category 4 PUs (n = 16 or 49%). The patients presenting with HAPUs were frequently re‐admitted, with 81% (n = 1132) of the primary cohort admitted to the hospital at least two times over the 41 study period. Of the HAPU patients, 78% (n = 1090) were admitted from home, with the remainder being admitted from residential care (n = 210 or 15%) or nursing homes (n = 98 or 7%). It was documented that 75% (n = 1049) of the HAPU patients were emergency admissions.

#### Comparison between patient sub‐populations

The demographics of the three sub‐groups revealed that those patients who had a Waterlow score >10 once during their hospital stay but did not develop a PU were statistically younger, demonstrated a lower peak Waterlow score, and a reduced length of stay compared to both the HAPU and CAPU groups (*P* < 0·001, for each case). Post hoc analysis revealed that the peak Waterlow scores and length of stay were significantly (*P* = 0·001) greater in the HAPU group than the no PU and CAPU groups (Figure 2). However, the age difference and frequency of risk assessment between CAPU and HAPU was not significant (*P* > 0·1). In addition, the trends were different with respect to PU category. Thus, while the median length of stay for HAPU group increased monotonically with PU category, there was little difference in the median length of stay for CAPU patients, across the four PU categories (Figure 2).

Close examination of Table 1 revealed the PU categories for both groups were different in distribution, with CAPUs presenting with a higher proportion (n = 916 or 70%) of category 1 PUs compared to HAPU (n = 696 or 50%). However, both sub‐groups had a small proportion of the most severe category 4 PUs (n = 20 and 32, or 2% in each case). The location of the PUs also differed between groups, with the CAPU ulcers predominantly being located at the sacrum (n = 920 or 73%) and buttocks (n = 251 or 19%). By contrast, patients with HAPUs had a larger proportion (n = 376 or 28%) located at the heels (Table 1).

## Discussion

This retrospective evaluation of data collected by single observer included 46,129 patients admitted to a District General hospital over a 41 month period. Of these patients, 14% were at risk of PUs according to the Waterlow risk assessment scale (score >10) at some point during their hospital stay. Patients at risk who did not acquire a PU were younger in age, tended to stay for a shorter period in hospital and were less likely to be re‐admitted during the study period than those with a CAPU or HAPU. The data also revealed that HAPU patients had a longer length of stay than CAPU for all categories of PU and there were also some distinct differences in the PU location.

The similar prevalence values for CAPU and HAPU, namely 2·7% and 3·0% respectively, concurs with that generally reported in the literature (Lahmann *et al*. 2006, Barrois *et al*. 2008, Phillips & Buttery 2009). However, some studies have reported contrasting findings, for example, a cross‐sectional study in Sweden reported the prevalence of HAPU was much higher than CAPU (11·6% vs. 3·3%). With respect to PU categories, this study demonstrated similar findings to those reported in the literature, namely over 50% of the pressure ulcers are category 1, and a significant proportion of PU categories 3–4 affect the heels and sacrum (Gunningberg *et al*. 2011). This study also revealed a disparity in hospital length of stay between CAPU and HAPU patients (Table 1), which, for all PU categories, revealed a lower average length of stay in the former patients (Figure 2), particularly pronounced when considering PU categories 3 and 4. There was also a difference in the incremental changes in length of hospital stay with PU category, with HAPUs hospital length of stay increasing monotonically with pressure ulcer category, while CAPU length of stay did not differ across the categories. Therefore, present results indicate that when assessing the socio‐economic impact of pressure ulcers using factors such as length of stay, patients with CAPU and HAPU should be treated as separate patient groups. Further research is clearly needed to identify the causal significance of PU origin with regard to hospital length of stay.

Although preventative strategies, in the form of pressure redistributing support surfaces, were administered to patients at PU risk, this was not implemented within 24 hours in a substantive proportion of HAPU (27%) and CAPU (19%) cases. The timing of support surface provision is a source of current debate with literature (McInnes *et al*. 2012). However, the findings from this study clearly indicate that the timing of support surface provision is not optimal for those who are admitted with or develop a PU during their stay. This study also revealed that a large proportion of the HAPU and CAPU patients (77‐78%) were readmitted to hospital multiple times over the 41 month study period. This readmission rate is higher than that previously reported, with a recent systematic review highlighting rates between 40‐50% (García‐Pérez *et al*. 2011). The high number of re‐admissions during this study may have been a consequence of the healthcare provision for the Island community, with the hospital being the main source of provision in the locality. However, this finding is worthy of further exploration.

The major limitation of the protocol adopted in this study was the reliance on the accuracy and completeness of the reporting forms. To minimize the limitations associated with the retrospective approach data was collected from a single observer enhancing internal consistency. The documented information included a finite number of clinical factors which were used in the analysis. Preventative strategies such as, for example, patient repositioning were not reported. Clearly, the quality of the documentation will have a large effect on the accuracy of the data that is collected (Gunningberg *et al*. 2000). Other important limitations include the use of peak Waterlow scores for each patient during their hospital stay. This could have omitted useful temporal changes in PU risk. In addition, the causality of increased length of stay and re‐admission rates is complex, the present data do not take into account many of the factors which could account for this, for example, comorbidities. The relationship between pressure ulcer status and length of stay/readmission rates, requires further investigation to account for all the confounding factors and their potential interactions.

The results from this study are important for clinical practice as they reveal some significant differences regarding the severity and location of PUs between those admitted with and those who develop a PU during admission. For example, heel PUs accounted for 28% of all HAPUs compared to 6% of CAPUs. The high incidence of hospital acquired heel PUs indicates that preventative management, in addition to provision of support surfaces, is needed specifically for vulnerable heels in the inpatient setting. There are also some significant gaps in practice when comparing to international standards of care (National Pressure Ulcer Advisory Panel, European Pressure Ulcer Advisory Panel and Pan Pacific Pressure Injury Alliance, 2014), with delays in the provision of pressure redistribution surfaces and too infrequent assessments of risk. The significant increase in hospital length of stay for the HAPU group will also have a financial impact on the healthcare provider, with a hospital bed estimated to cost £200 per day in the UK (NHS England, 2015). The presented study also revealed a higher prevalence of CAPU and HAPU in patients that were admitted from a nursing or residential home when compared to those who were at risk but did not acquire a pressure ulcer during their stay (19‐22% vs. 7%). Indeed, there is compelling evidence from the literature of high PU prevalence rates in the nursing home settings across Europe (Tannen *et al*. 2008). A greater understanding of how patients are managed in the hospital setting and the influence of admission location is worthy of further investigation.

## Conclusion

This study has shown that patients admitted to a General District hospital with a PU (CAPU) or acquire a PU during their inpatient stay (HAPU) are older and have an extended length of stay than those at risk who do not develop a PU. This study has also shown that a proportion of HAPU and CAPU patients do not receive a pressure redistributing support surface within 24 hours of being defined at risk of PUs. The retrospective evaluation of patient records also revealed that HAPU patients have an extended length of hospital stay and have a higher proportion of pressure ulcers in the heels compared to CAPU patients. In addition, those with CAPU or HAPU were more likely to have been admitted from a nursing or residential home setting. Further prospective studies are required to investigate the care pathway factors which can influence CAPU and HAPU patients and future healthcare cost models need to address the differences observed between these patient sub‐populations.

## Author contributions

Worsley, Bader and Schoonhoven contributed to the study conception/design; drafting of manuscript; supervision; statistical expertise. Smith was responsible for data collection/analysis and drafting of manuscript.

All authors have agreed on the final version and meet at least one of the following criteria [recommended by the ICMJE (http://www.icmje.org/recommendations/)]:

- substantial contributions to conception and design, acquisition of data, or analysis and interpretation of data;
- drafting the article or revising it critically for important intellectual content.

## References

[nop250-bib-0001] Agency for Healthcare Research and Quality (AHRQ) (2011) Preventing Pressure Ulcers in Hospitals: A Toolkit for Improving Quality [Online]. Rockville, MD Retrieved from http://www.ahrq.gov/professionals/systems/hospital/pressureulcertoolkit/index.html on 19 June 2015.

[nop250-bib-0002] Barrois B. , Labalette C. , Rousseau P. , Corbin A. , Colin D. & Saumet J.L. (2008) A national prevalence study of pressure ulcers in French hospital inpatients. Journal of Wound Care 17, 378–9. 10.12968/jowc.2008.17.9.30934 18833894

[nop250-bib-0003] Brown C.J. & Flood K.L. (2013) Mobility limitation in the older patient: a clinical review. JAMA 310, 1168–1177. 24045741 10.1001/jama.2013.276566

[nop250-bib-0004] Coleman S. , Gorecki C. , Nelson A. , Closs J.S. , Defloor T. , Halfens R. , Farrin A. , Brown J. , Schoonhoven L. & Nixon J. (2013) Patient Risk Factors for pressure ulcer development: systematic Review. International Journal of Nursing Studies 50, 974–1003. 23375662 10.1016/j.ijnurstu.2012.11.019

[nop250-bib-0005] Dealey C. , Posnett J. & Walker A. (2012) The cost of pressure ulcers in the United Kingdom. Journal of Wound Care 21, 261–266. 22886290 10.12968/jowc.2012.21.6.261

[nop250-bib-0006] Department of Health (2010) Essence of Care 2010; Benchmarks for Prevention and Management of Pressure Ulcers. The Stationery Office, UK.

[nop250-bib-0007] EPUAP‐NPUAP (2009) Prevention and Treatment of Pressure Ulcers: Quick Reference Guide. European Pressure Ulcer Advisory Panel and National Pressure Ulcer Advisory Panel, Washington, DC.

[nop250-bib-0009] Gallagher P. , Barry P. , Hartigan I. , McCluskey P. , O'Connor K. & O'Connor M. (2008) Prevalence of pressure ulcers in three university teaching hospitals in Ireland. Journal of Tissue Viability 17, 103–109. 18378140 10.1016/j.jtv.2007.12.001

[nop250-bib-0010] García‐Pérez L. , Linertová R. , Lorenzo‐Riera A. , Vázquez‐Díaz J. R. , Duque‐González B. & Sarría‐Santamera A. (2011) Risk Factors for Hospital Readmissions in Elderly Patients: A Systematic Review. 104(8), 639–651. 10.1093/qjmed/hcr070 21558329

[nop250-bib-0011] Gunningberg L. , Lindholm C. , Phd M.C. & Phd P.‐O.S. (2000) The development of pressure ulcers in patients with hip fractures: inadequate nursing documentation is still a problem. Journal of Advanced Nursing 31, 1155–1164. 10840249

[nop250-bib-0012] Gunningberg L. , Stotts N.A. & Idvall E. (2011) Hospital‐acquired pressure ulcers in two Swedish County Councils: cross‐sectional data as the foundation for future quality improvement. International Wound Journal 8, 465–473. 21722316 10.1111/j.1742-481X.2011.00818.x PMC7950814

[nop250-bib-0013] Gunningberg L. , Hommel A. , Bååth C. & Idvall E. (2013) The first national pressure ulcer prevalence survey in county council and municipality settings in Sweden. Journal of Evaluation in Clinical Practice 19, 862–867. 22640165 10.1111/j.1365-2753.2012.01865.x

[nop250-bib-0014] Keelaghan E. , Margolis D. , Zhan M. & Baumgarten M. (2008) Prevalence of pressure ulcers on hospital admission among nursing home residents transferred to the hospital. Wound Repair and Regeneration 16, 331–336. 18471251 10.1111/j.1524-475X.2008.00373.x PMC2839543

[nop250-bib-0015] Lahmann N. , Halfens R. & Dassen T. (2006) Pressure ulcers in German nursing homes and acute care hospitals: prevalence, frequency, and ulcer characteristics. Ostomy Wound Management 52, 20–33. 16464992

[nop250-bib-0016] McInnes E. , Jammali‐Blasi A. , Bell S.S. , Dumville J. & Cullum N. (2012) Preventing pressure ulcers – are pressure‐redistributing support surfaces effective? A Cochrane systematic review and meta‐analysis. International Journal of Nursing Studies 49, 345–59. 22104042 10.1016/j.ijnurstu.2011.10.014

[nop250-bib-0017] Moore Z. & Cowman S. (2012) Pressure ulcer prevalence and prevention practices in care of the older person in the Republic of Ireland. Journal of Clinical Nursing 21, 362–371. 21733017 10.1111/j.1365-2702.2011.03749.x

[nop250-bib-3000] National Pressure Ulcer Advisory Panel, European Pressure Ulcer Advisory Panel and Pan Pacific Pressure Injury Alliance . (2014) Prevention and Treatment of Pressure Ulcers: Quick Reference Guide. Emily Haesler (Ed.). Cambridge Media: Osborne Park, Western Australia.

[nop250-bib-0018] NHS England (2015) National Tariff Payment System MONITOR, London, UK.

[nop250-bib-0019] Oot‐Giromini B.A. (1993) Pressure ulcer prevalence, incidence and associated risk factors in the community. Advances in Skin & Wound Care 6, 24–35. 8286017

[nop250-bib-0020] Phillips L. & Buttery J. (2009) Exploring pressure ulcer prevalence and preventative care. Nursing Times 105, 34–6. 19480167

[nop250-bib-0021] Severens J.L. , Habraken J.M. , Duivenvoorden S. & Frederiks C.M.A. (2002) The cost of illness of pressure ulcers in the Netherlands. Advances in Skin and Wound Care 15, 72–77. 11984050 10.1097/00129334-200203000-00008

[nop250-bib-0022] Smith S.M. , Soubhi H. , Fortin M. , Hudon C. & O'Dowd T. (2012) Managing Patients with Multimorbidity: Systematic Review of Interventions in Primary Care and Community Settings. 345: e5205. 10.1136/bmj.e5205 PMC3432635 22945950

[nop250-bib-0023] Spilsbury K. , Nelson A. , Cullum N. , Nixon J. & Mason S. (2007) Pressure ulcers and their treatment and effects on quality of life: hospital inpatient perspectives. Journal of Advanced Nursing 57, 494–504. 17284276 10.1111/j.1365-2648.2006.04140.x

[nop250-bib-0024] Tannen A. , Dassen T. & Halfens R. (2008) Differences in prevalence of pressure ulcers between the Netherlands and Germany – associations between risk, prevention and occurrence of pressure ulcers in hospitals and nursing homes. Journal of Clinical Nursing 17, 1237–1244. 18416798 10.1111/j.1365-2702.2007.02225.x

[nop250-bib-0025] Theisen S. , Drabik A. & Stock S. (2012) Pressure ulcers in older hospitalised patients and its impact on length of stay: a retrospective observational study. Journal of Clinical Nursing 21, 380–387. 22150944 10.1111/j.1365-2702.2011.03915.x

[nop250-bib-0026] Vanderwee K. , Defloor T. , Beeckman D. , Demarré L. , Verhaeghe S. , Van Durme T. & Gobert M. (2011) Assessing the adequacy of pressure ulcer prevention in hospitals: a nationwide prevalence survey. BMJ Quality & Safety 20, 260–267. 10.1136/bmjqs.2010.043125 21209147

[nop250-bib-0027] VanGilder C. , Amlung S. , Harrison P. & Meyer S. (2009) Results of the 2008–2009 internationa pressure ulcer prevalence™ survey and a 3‐year, acute care, unit‐specific analysis. Ostomy Wound Management 55, 39–45. 19934462

